# PGS and the Critical Decision on Transfer of Defective Embryos

**Published:** 2018

**Authors:** Mohammad Reza Sadeghi

Today, pre-implantation genetic diagnosis/screening (PGD/PGS) tests are a key part of reproductive medicine in most countries. These techniques have been designed for a variety of purposes, including identification of embryos affected or carriers of monogenic diseases, identification of aneuploid embryos or selection of embryos with specific gender. Technical updates and improvements of PGD/PGS were done over time. According to CDC statistics, about 4–6% of ART cycles are candidates for PGS annually and there is evidence on the increase in request (1). Insomuch that the first use of the FISH technique in 1900 created new hopes for elimination of aneuploid embryos in the IVF cycles. For twenty years, the FISH technique was the undeniable method for PGS of embryos in IVF centers. But a large number of studies in the first years of recent decade failed to show the benefits of FISH/PGS for improvement of pregnancy rate in IVF cycles. At the beginning of recent decade, new tools and technologies of CGH array, SNP array, quantitative PCR and more recently NGS were developed and utilized for improvement of PGS results. This replacement has many advantages over older tools. Certain features of recent PGS techniques allow simultaneous evaluation of 23 pairs of chromosomes, the use of trophectoderm biopsy and more cells for evaluation and also the possibility of verifying biopsied blastomeres. Despite the development and effectiveness of new techniques and their ability to identify the maximum euploid embryos, a limited number of embryos is reported with chromosomal mosaicism due to using just 5–10 cells for genetic assessment (2). The rate of mosaicism in the new technologies is significantly reduced in comparison with FISH (3–5% versus 50%). If mosaic embryos at day 3 are cultured to day 5, approximately 50% of them would be self-corrected to euploid blastocysts, but a number of embryos are still reported as mosaic in PGS results. The mosaicism reports have formed a challenge in application of genetic testing of IVF embryos and subsequently patients counseling. It is always difficult to make decisions about the transfer of these embryos, particularly in cases where all embryos are reported mosaic or aneuploid and the couple cannot try for another cycle (3). The current practices predominantly transfer euploid embryos; however, a lot of data support the birth of healthy babies from mosaic embryos. Therefore, gynecologists, embryologists, geneticists, and patients applying for PGD/PGS in IVF clinics are faced with new challenges for mosaicism in genetic counseling. Therefore, it is imperative that all specialties related to the provision of these services collaborate to plan a comprehensive patient consultation protocol pre and post PGS. The counseling strategy and providing the correct information has a critical role in patient’s decision and avoidance of undesired outcomes for couples and service providers in the future. One of the main duties of pre-PGS counseling is to set realistic expectations for couples about PGS. Counseling sessions should include discussions on the challenges associated with PGS, the frequency of mosaicism regarding couple’s age, the false positive/negative results and patients should be briefed on the limited data about its effectiveness and also the interpretation of PGS results. Precise and comprehensive counseling may dissuade the couples who have little information or misconceptions about potentials of PGS (4).

But in cases where couples inevitably request the transfer of mosaic embryos, the importance of post PGS consultation is more critical. The data discussed at this level should be different from pre-PGS counseling. The couples should be aware of the potential risks encountered with implantation of mosaic embryos, their lower implantation rate and higher abortion rate, the risk of fetal demise or uniparental disomy, the emotional and financial risk and also the couples should be informed that most of associated risks following mosaic embryos transfer are still unknown. Therefore, pregnancies following transfer of mosaic embryos should be referred for prenatal screening to detect any aneuploidy in the fetus (4). Recently, the International Preimplantation Genetics Association has issued a guideline for the transfer of mosaic embryos. According to this guideline, transfer of euploid embryos should be preferred to mosaic ones and mosaic monosomies to mosaic trisomies. If we have to transfer a mosaic trisomy embryo, mosaic trisomies of 1, 3, 4, 5, 6, 8, 9, 10, 11, 12, 17, 19, 20, 22, X, and Y are preferred over mosaic trisomies of 2, 7, 13, 14, 15, 16, 18, and 21 (3).

In addition to the challenge of mosaic in PGS, there is a similar and even more important challenge for PGD in which due to economic, ethical, religious issues or couples' request, physcians are forced to transfer carrier embryos or the ones affected with single-gene disorders. There are several considerations on the request for transfer of genetically defective embryos; the couple’s reproductive autonomy and liberty, physician’s professional conscience, professional norms, laws, and practices, health of born children and psychological and medical consequences for pregnant spouse are the typical issues. Therefore, making decisions on transfer of probably defective embryos is not so simple and straightforward and consequently before the final decision, all the above considerations should be taken into consideration (1). In addition, acoording to the recent committee opinion of American Society for Reproductive Medicine (ASRM) and the Society for Assisted Reproductive Technology (SART) the value of PGS for preimplantation screening of euploid and aneuploid embryos are not conclusive, because the previuose studies have important limitations and more studies need to be perform in future (5).

Since transfer of defective embryos is associated with numerous medical, psychological, and socio-economic consequences, until obtaining further information in this area, it is recommended the couples try another cycle to find healthy euploid embryos instead of tranfer of mosaic or genetically defective embryos.
